# Unveiling the Potential of Endophytic *Bacillus amyloliquefaciens* LJ1 from Nanguo Pear: A Genomic and Functional Study for Biocontrol of Post-Harvest Rot

**DOI:** 10.3390/foods14173020

**Published:** 2025-08-28

**Authors:** Zilong Li, Jiamin Jiang, Keyu Sun, Shuhong Ye

**Affiliations:** SKL of Marine Food Processing & Safety Control, School of Food Science and Technology, Dalian Polytechnic University, Dalian 116034, China; a584317213@163.com (Z.L.); m18378454350@163.com (J.J.); keyusun0523@163.com (K.S.)

**Keywords:** *Bacillus amyloliquefaciens* LJ1, functional analyses, whole-genome, safety assessments, biocontrol

## Abstract

*Bacillus amyloliquefaciens* is a well-recognized biocontrol agent and plant growth promoter. This study characterized the endophytic *B. amyloliquefaciens* LJ1, isolated from Nanguo pear fruit, through whole-genome sequencing and functional analyses. The *B. amyloliquefaciens* LJ1 genome (3,947,365 bp, 46.48% GC content) encodes 3757 protein-coding sequences. Genomic analysis revealed diverse carbohydrate-active enzymes (CAZymes) and 12 secondary metabolite biosynthetic gene clusters, including those potentially producing surfactin, fengycin, bacillibactin, and bacilysin. Safety assessments, including hemolysis, indole production, biogenic amine production, and a 21 day mice-feeding trial, indicated no adverse effects, suggesting *B. amyloliquefaciens* LJ1 is non-pathogenic. In vitro assays demonstrated significant inhibitory activity against *Penicillium expansum*, a major post-harvest pathogen, by suppressing spore germination and germ-tube elongation. These results suggest that *B. amyloliquefaciens* LJ1 possesses significant biocontrol potential and could be a promising agent for sustainable disease management in Nanguo pear and potentially other crops.

## 1. Introduction

The plant microbiome, a complex tapestry of microbial communities intimately associated with plants, is now recognized as a crucial determinant of plant health, productivity, and resilience. Among these microorganisms, endophytic bacteria, those colonizing internal plant tissues without causing apparent harm, have emerged as key players in plant–microbe interactions, conferring a broad spectrum of beneficial effects to their hosts [1]. These benefits encompass enhanced nutrient acquisition through nitrogen fixation and phosphate solubilization, promotion of plant growth via phytohormone production, and improved protection against various phytopathogens [2,3]. Exploiting the inherent power of these endophytic bacteria to fortify plant health and combat disease is a pivotal step toward sustainable and resilient agricultural systems.

The remarkable ability of endophytic bacteria to shield plants from disease stems from their diverse and sophisticated arsenal of defensive mechanisms. These include direct antagonism through the production of potent antimicrobial compounds such as bacteriocins, lipopeptides, and volatile organic compounds; fierce competition for vital nutrients and colonization sites; and the strategic induction of host plant systemic resistance [4,5,6]. For instance, certain species of endophytic *Bacillus* produce lipopeptides like surfactin and fengycin, which disrupt the structural integrity of fungal pathogen cell membranes, effectively inhibiting their growth and proliferation [7]. Complementarily, other endophytes generate volatile organic compounds, such as α-pinene, methyl salicylate and 2-ethyl hexanol, capable of directly inhibiting pathogen development or triggering a robust ISR response in plants, thereby enhancing resistance against subsequent attacks [8,9]. The complexity and sophistication of these interactions underscore the potential of endophytic bacteria as sustainable alternatives to conventional disease management strategies.

*Bacillus amyloliquefaciens*, a ubiquitous and versatile Gram-positive bacterium, is widely recognized for its remarkable biocontrol and plant growth-promoting capabilities [10]. This species demonstrates broad-spectrum antagonism against diverse phytopathogens, including fungi, bacteria, and viruses [11]. Its biocontrol efficacy is largely mediated by the synthesis of bioactive secondary metabolites, such as lipopeptides, polyketides, and lytic enzymes capable of degrading pathogen cell walls [12,13]. Additionally, *B. amyloliquefaciens* promotes plant growth via the secretion of phytohormones and enhancement of nutrient acquisition through phosphate solubilization and biological nitrogen fixation [14]. Its adaptability and multifaceted beneficial properties make *B. amyloliquefaciens* an attractive candidate for the development of sustainable agricultural practices.

Endophytic community composition and function are significantly influenced by plant species and environmental conditions, resulting in the selection of specialized strains exhibiting host-specific interactions and adaptation to particular ecological niches [15]. Nanguo pear, valued for its characteristic flavor and nutritional composition, faces significant production constraints due to diseases such as pear scab, black spot, and blue mold, resulting in substantial yield and quality losses [16]. Investigating the endophytic bacterial communities inhabiting Nanguo pear fruits offers a valuable opportunity to identify novel biocontrol agents specifically adapted to this crop and its associated disease challenges. Isolating and characterizing *B. amyloliquefaciens* from this niche could uncover strains with enhanced biocontrol activity, improved colonization competence, and unique mechanisms for suppressing Nanguo pear diseases. Therefore, this study used whole-genome sequencing analysis to deeply investigate the metabolic system and pathogenicity mechanisms of the endophytic *B. amyloliquefaciens* LJ1 from Nanguo pear and to comprehensively analyze the biological characteristics of this strain. Additionally, the safety of the strain was studied, including hemolytic activity, indole production, and biogenic amine research. The bacterial effects on the physiological and pathological states of mice were assessed through mouse gastric gavage toxicity tests to confirm its feasibility and safety in applications. Finally, in vitro inhibition experiments against *Penicillium expansum* were conducted to provide a scientific basis for the potential application of this strain.

## 2. Materials and Methods

### 2.1. Strains and Culture Conditions

*B. amyloliquefaciens* LJ1 (NCBI accession number: CP195073) was previously isolated from Nanguo pear fruit in our laboratory. *Penicillium expansum* was obtained from our laboratory’s stock culture collection. *B. amyloliquefaciens* LJ1 and *P. expansum* were cultured in luria–bertani (LB) and potato dextrose agar (PDA) media, respectively, and incubated at 28 °C for subsequent experiments.

### 2.2. DNA Extraction and Whole-Genome Sequencing

*B. amyloliquefaciens* LJ1 was cultured in LB broth at 28 °C with shaking at 170 rpm for 12 h. Genomic DNA was extracted using the TIANamp Bacteria DNA Kit (Tiangen Biotech Co., Ltd., Beijing, China) according to the manufacturer’s instructions. The quantity and quality of the extracted DNA were assessed using a NanoDrop 2000 spectrophotometer (Thermo, Waltham, MA, USA) and agarose gel electrophoresis. Whole-genome sequencing was performed by Shanghai Meiji Biomedical Technology Co., Ltd. (Shanghai, China) using both Illumina HiSeq × 10 and PacBio platforms [16].

### 2.3. Genome Assembly and Optimization

Raw reads obtained from the Illumina platform underwent quality control using Fastp 0.20.0 to trim low-quality reads and adapter sequences, yielding clean data. The quality of the clean reads was then assessed using SOAPdenovo2 and Meryl 1.3 to evaluate GC content, genome repetitiveness, genome size, and the presence of plasmid sequences. The data that passed quality filtering were visualized by generating GC depth distribution plots and K-mer frequency distribution plots. De novo genome assembly was initially performed using the short-read assembler SOAPdenovo2 to generate scaffolds [17]. Final genome assembly was accomplished using Unicycler v0.4.8, incorporating long reads from the third-generation PacBio sequencing [18]. The resulting assembly was polished using Pilon v1.22 to correct any remaining base-calling errors.

### 2.4. Genome Annotation

Coding sequences (CDSs) within the chromosomal region were predicted using Glimmer 2, while CDSs within plasmid regions were predicted using GeneMarkS 4.3. This process yielded nucleotide and amino acid sequences of predicted functional genes [19,20]. Transfer RNA (tRNA) genes were predicted using tRNAscan-SE v2.0, and ribosomal RNA (rRNA) genes were identified using Barrnap 0.9. The identified core genes were validated using BLAST 2.3.0. Functional information was obtained through comparative analysis against multiple authoritative databases, including the non-redundant protein (NR) database, Swiss-Prot, Pfam, the Clusters of Orthologous Groups (COG) database, the Gene Ontology (GO) database, and the Kyoto Encyclopedia of Genes and Genomes (KEGG) database [21]. A systematic annotation was performed based on sequence homology, domains, and functional classifications derived from the comparisons with these databases. This allowed for the identification of key enzymes and metabolic pathways, providing a foundation for gene function determination.

### 2.5. Analysis of Metabolic Systems

To explore the functional diversity of carbohydrate-active enzymes (CAZyme), the assembled genome was analyzed using the CAZyme database [22]. The CAZyme database provides a comprehensive classification system for identifying and classifying enzymes involved in carbohydrate metabolism, including glycoside hydrolases (GHs), glycosyltransferases (GTs), polysaccharide lyases (PLs), carbohydrate esterases (CEs), carbohydrate-binding modules (CBMs), and auxiliary activities (AAs). This analysis aimed to elucidate the comprehensive functional role of CAZymes in carbohydrate metabolism within this strain, particularly their involvement in the degradation, modification, and formation of glycosidic bonds. To identify potential biosynthetic gene clusters for secondary metabolites, the genome was analyzed using the antiSMASH database [23].

### 2.6. Pathogenicity Analysis

The bacterial genome was analyzed using a variety of databases and software tools, including VFDB (20230407), CARD v3.2.6, PHI 4.9, ResFinder 4.3.1, KEGG, TCDB v20200917, Pfam 33.1, Diamond 0.8.35, ResFinder_v4.1.0, SignalP 4.1, TMHMM 2, and HMMER3, to investigate virulence genes, antimicrobial resistance genes, antimicrobial resistance genes (ResFinder), secretion systems, secreted proteins, transporter proteins and transmembrane proteins. These methods enabled a comprehensive analysis of the bacterial pathogenicity mechanisms, including the expression of virulence factors, the development of antimicrobial resistance, interactions with the host, and critical secretion and transport systems. These analyses provided important molecular-level insights into the pathogenicity and adaptability of the bacterium.

### 2.7. Safety Assessment

The hemolytic activity of the strain was assessed using Columbia blood agar, following previously described methods [24]. The activated strain was inoculated onto Columbia blood agar supplemented with 5% (*v*/*v*) human blood (5 g/100 mL) and incubated at 28 °C for 48 h. The hemolytic type was determined by observing the hemolysis pattern surrounding the colonies. *Escherichia coli* ATCC 25922 was used as a positive control to ensure the accuracy and reliability of the results.

The ability of the strain to produce indole during cultivation was determined using an indole assay [25]. The test strain was inoculated into tryptone water broth and incubated at 28 °C for 48 h, after which 8–10 drops of Kovac’s reagent were added to the culture. Subsequent color changes were carefully observed. *E. coli* ATCC 25922 was used as a positive control to validate the efficacy of the procedure and reagents.

The ability of the strain to produce specific biogenic amines during cultivation was determined by assessing amino acid decarboxylase activity [26]. Specifically, we assessed the decarboxylation of tyrosine, histidine, lysine, and ornithine, which can lead to the production of tyramine, histamine, cadaverine, and putrescine, respectively. These biogenic amines are often associated with food spoilage and, at high concentrations, can pose health risks. The *B. amyloliquefaciens* LJ1 strain was inoculated into decarboxylase detection medium (5 g^−L^ peptone, 1 g^−L^ glucose, 0.02% bromothymol blue) supplemented with 0.1% (*w*/*v*) of a specific L-amino acid (L-tyrosine, L-histidine, L-lysine, or L-ornithine). All tubes were overlaid with sterile mineral oil to create anaerobic conditions, favoring decarboxylase activity. The tubes were then incubated at 28 °C for 48 h. Decarboxylation was assessed by observing color changes in the medium. A positive result (amine production) was indicated by a shift to a purple or violet color due to the increase in pH from amine production. A negative result was indicated by the medium remaining yellowish or unchanged (indicating no decarboxylation). *E. coli* ATCC 25922 was used as a positive control for the decarboxylation of lysine to cadaverine to ensure the accuracy of the method and the viability of the decarboxylase medium.

### 2.8. Animal Experiment Design

C57BL/6 male mice (8 weeks old) were obtained from Liaoning Changsheng Biotechnology Co., Ltd. (Changchun, China) and allowed a one-week acclimation period before the experiment. All procedures were approved by the Experimental Animal Ethics Committee of the National Engineering Research Center of Seafood at Dalian Polytechnic University (SYXK2017-0005). Thirty mice were randomly divided into two groups: a healthy control group (sterile physiological saline, control) and a *B. amyloliquefaciens* LJ1 treatment group (1.5 × 10^9^ CFU/mL, LJ1). Each group received a gavage treatment once every three days; the gavage volume was 0.5 mL per mouse per treatment, and the treatment was continued for 21 days. Mice body weights were recorded every three days during the treatment period. Mice were fasted for 12 h before termination by blood collection via eyeball extirpation. All animal procedures were performed in accordance with the European Parliament and Council Directive 2010/63/EU on the protection of animals used for scientific purposes [27].

For the liver, kidneys, and other major organs of the mice, the organs were rinsed with physiological saline to remove blood and residual tissue. After rinsing, excess moisture was removed from the surface of each organ with filter paper, and the wet weight of each organ was measured using an electronic balance. The organ weight coefficient was calculated using the following formula: Organ Weight Coefficient = (Organ Wet Weight/Mice Body Weight) × 100%. The collected tissues were sectioned at 5 μm thickness, stained with hematoxylin and eosin (H & E), and observed using a Nikon Ti-S fluorescence microscope [28].

### 2.9. Effects of Bacillus amyloliquefaciens LJ1 on Penicillium expansum In Vitro

*P. expansum* spores were spread on PDA plates. After 24 h of incubation, a 15 mm diameter fungal disk was excised from the plate using a hole puncher and placed in the center of a new PDA plate. *B. amyloliquefaciens* LJ1 was inoculated 3 cm from both sides of the *P. expansum* disk. After incubation at 28 °C for 72 h, the colony diameters were measured.

*B. amyloliquefaciens* LJ1 was diluted with sterile water to prepare a series of bacterial suspensions with a concentration range of 1 × 10^9^ CFU/mL. One milliliter of each diluted *B. amyloliquefaciens* LJ1 suspension (equal volume of sterile water for the control) was added to 1 L of PDB medium containing *P. expansum* spores at a concentration of 1 × 10^6^ spores/mL. The cultures were incubated at 25 °C for 48 h. Spore germination and germ-tube length of *P. expansum* were observed under an optical microscope at 6, 12, 18, 24, and 48 h.

### 2.10. Effects of Bacillus amyloliquefaciens LJ1 on Penicillium expansum In Vivo

Select Nanguo pears with intact surfaces, using a hole puncher, create wounds on the surface of each pear with a diameter of 5 mm and a depth of 3 mm. Inject 50 μL of *B. amyloliquefaciens* LJ1 suspension (10^9^ CFUmL^−1^) into each wound (for the control group, inject 50 μL of sterile water into the wound). After treatment, store all groups at 20 °C for 3 h, followed by an injection of 50 μL of *P. expansum* suspension into each wound. Place the sample in a room temperature environment and observe the occurrence of diseases for 7 days.

### 2.11. Statistical Analysis

Data were analyzed using SPSS Statistics 19.0. The effects of treatments were assessed using one-way analysis of variance (ANOVA), and Duncan’s multiple range test was used for comparisons of means. A significance level of *p* < 0.05 was used for all statistical tests.

## 3. Results and Discussion

### 3.1. Genome Assessment

**Figure 1:**
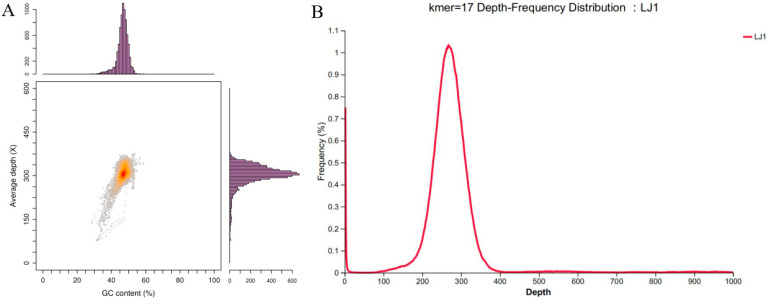
Genomic evaluation. (**A**) GC depth distribution analysis (Depth of staining indicates enrichment); (**B**) K-mer frequency distribution analysis.

The quality of the *B. amyloliquefaciens* LJ1 genome assembly was evaluated through GC content analysis, sequencing depth assessment, and k-mer frequency distribution (Figure 1). The GC content exhibited a unimodal distribution centered around 45%, consistent with previously reported values for *B. amyloliquefaciens* species [29]. This suggests a homogenous genomic composition and the absence of recent large-scale horizontal gene transfer events. A high average sequencing depth of approximately 300× indicated a robust dataset, minimizing the potential for sequencing errors and ensuring high confidence in base calling. Adequate sequencing depth is crucial for accurate variant identification and reliable genome annotation. K-mer analysis (k = 17) revealed a sharp, single peak at a depth of approximately 300, indicating low genome duplication, minimal contamination, and effective resolution of repetitive sequences during assembly. The uniform k-mer distribution suggests low redundancy and a minimal level of contamination. The absence of multiple peaks indicates that the assembly process successfully resolved repetitive regions and accurately reconstructed the genome. In summary, the results demonstrate that the sequencing data for *B. amyloliquefaciens* LJ1 is of high quality and the genome assembly is accurate and reliable, providing a solid foundation for studying the genetic characteristics and functional mechanisms of *B. amyloliquefaciens* LJ1.

### 3.2. Genome Assembly and Prediction

The *B. amyloliquefaciens* LJ1 genome was assembled into a single chromosome with a total length of 3,947,365 bp and a GC content of 46.48% (Table 1). No plasmids were detected. Genome annotation predicted 3757 protein-coding sequences (CDSs), totaling 3,485,559 bp, with an average length of 927.75 bp and a gene density of 0.95 kb. The GC content was 47.32% in gene regions and 40.17% in intergenic regions, with 88.30% of the genome comprising coding sequences (Table 2). In addition to CDSs, the genome contained 95 tRNA genes (20 different types) and 30 rRNA genes (10 each of 16S, 23S, and 5S rRNA). Further annotation revealed 31 housekeeping genes, 81 sRNAs, 70 tandem repeat sequences (0.43% of the genome), 16 SINEs, 25 LINEs, 2 LTRs, and 7 DNA transposons.

**Figure 2:**
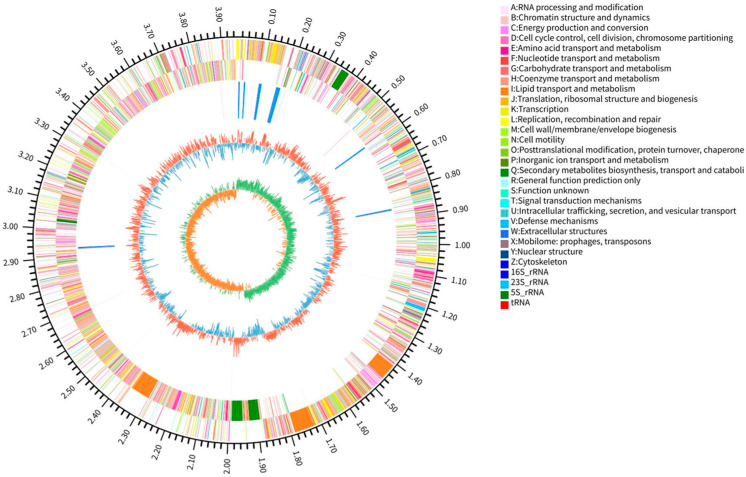
Circos genome circle plot. The outermost circle of the circle plot indicates the size of the genome; the second and third circles represent the CDS on the positive and negative strands, respectively, with different colors indicating the different COG functional classifications of the CDS; the fourth circle represents rRNA and tRNA; the fifth circle represents the GC content, with the outward red part indicating that the GC content of this region is higher than the average GC content of the whole genome, and the higher the peak, the greater the difference from the average GC content; the inward blue part indicates that the GC content of this region is lower than the average GC content of the whole genome, and the higher the peak, the greater the difference from the average GC content.

A comprehensive visualization of the overall genome organization of *B. amyloliquefaciens* LJ1 was achieved using a Circos plot (Figure 2), providing a framework for understanding relationships between various genomic features. The plot displayed the following information, moving from the outermost to the innermost circle: genome base pair size; protein-coding sequences (CDSs) located on the forward and reverse strands, with different colors representing different Clusters of Orthologous Groups (COG) functional categories; ribosomal RNA (rRNA) and transfer RNA (tRNA) gene locations; GC content, with outward-pointing red peaks indicating regions with GC content higher than the genome average and inward-pointing blue peaks indicating regions with GC content lower than the genome average; and GC skew, calculated as (G − C)/(G + C), used to infer the leading and lagging strands during replication and to approximate the origin and terminus of replication. The genome size and GC content were both within the typical range for *B. amyloliquefaciens*, consistent with values reported in the literature [29]. However, the absence of plasmids does not exclude their transient presence or the presence of undetected cryptic plasmids contributing to specific traits, such as antibiotic resistance or tolerance [30]. The abundance of CDSs, tRNAs, and rRNAs suggests a robust protein synthesis capacity and a potential for rapid adaptation to environmental changes. The distribution of COG functional categories highlighted the metabolic potential of *B. amyloliquefaciens* LJ1. The enrichment of genes involved in energy production and conversion, amino acid transport and metabolism, carbohydrate transport and metabolism, and the biosynthesis, transport, and catabolism of secondary metabolites suggests that *B. amyloliquefaciens* LJ1 possesses diverse mechanisms for interacting with its environment, including the production of antimicrobial compounds or the metabolism of plant-derived compounds. The abundance of genes associated with energy production, amino acid, and carbohydrate metabolism highlights the metabolic versatility of *B. amyloliquefaciens* LJ1 and its ability to thrive in diverse environments and utilize a wide range of nutrients. Notably, the presence of a substantial number of genes in the “secondary metabolite biosynthesis, transport, and catabolism” category indicates a significant capacity for *B. amyloliquefaciens* LJ1 to produce a variety of bioactive compounds, potentially playing roles in plant growth promotion and biocontrol [31]. The presence of sRNAs and mobile genetic elements further suggests a potential for genomic plasticity and adaptation, enabling *B. amyloliquefaciens* LJ1 to fine-tune its gene expression and metabolic pathways in response to environmental cues [32]. Overall, the genomic architecture of *B. amyloliquefaciens* LJ1 provides valuable insights into its potential functional capabilities. The combination of conserved genomic features with unique regulatory elements indicates a complex interplay between core metabolic functions and adaptive responses.

### 3.3. Gene Annotation

To comprehensively characterize the functional potential of *B. amyloliquefaciens* LJ1, we performed functional annotation of its genome by integrating information from multiple databases and classification schemes, including the NR database, Swiss-Prot, Pfam, COG, GO, and KEGG pathways.

**Figure 3:**
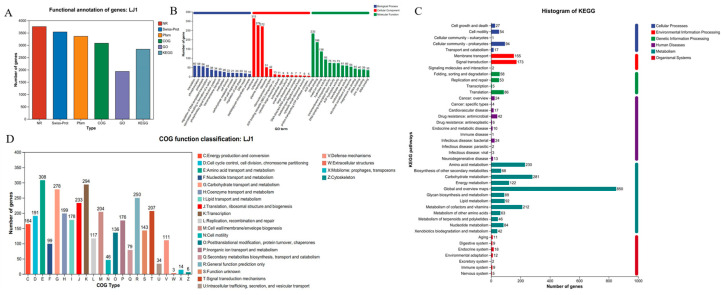
Gene annotation. (**A**) Summarize Comments; (**B**) GO annotation; (**C**) KEGG annotation; (**D**) COG annotations.

To comprehensively characterize the functional potential of *B. amyloliquefaciens* LJ1, we performed functional annotation of its genome by integrating information from multiple databases and classification schemes. Overall, the majority of predicted protein-coding genes showed significant similarity to sequences in the NR database (3757 genes), followed by Swiss-Prot (3535 genes) and Pfam (3345 genes) (Figure 3A), indicating a high degree of conservation with known proteins and protein domains. COG functional classification (Figure 3D) revealed that the most abundant categories were Translation, ribosomal structure and biogenesis (J, 294 genes), Posttranslational modification, protein turnover, chaperones (O, 136 genes), Carbohydrate transport and metabolism (G, 278 genes), Amino acid transport and metabolism (E, 308 genes), Energy production and conversion (C, 164 genes), and Transcription (K, 233 genes), suggesting active protein synthesis, maintenance, and energy production for survival and adaptation. GO enrichment analysis (Figure 3B) identified dominant terms in Biological Process as translation (60 genes), transport (59 genes), and phosphorylation (56 genes); in Cellular Component as membrane (276 genes), cytoplasm (172 genes), and ribosomes (51 genes); and in Molecular Function as ATP binding (233 genes), metal ion binding (196 genes), and DNA binding (136 genes), further highlighting LJ1’s transport capabilities and metabolic activity. KEGG pathway analysis (Figure 3C) revealed a significant number of genes involved in global and overview metabolic pathways (850 genes), carbohydrate metabolism (281 genes), amino acid metabolism (230 genes), and metabolism of cofactors and vitamins (212 genes), with notable involvement in secondary metabolite biosynthesis (68 genes) that might contribute to bio-control.

The integrated analysis of the functional annotation results, based on NR, Swiss-Prot, Pfam, COG, GO, and KEGG databases, provides a comprehensive understanding of the metabolic capabilities and potential biocontrol mechanisms of *B. amyloliquefaciens* LJ1. The high proportion of genes involved in carbohydrate and amino acid metabolism reflects the ability of this species to utilize diverse nutrient sources in the rhizosphere, supporting its robust growth and competitive interactions with other microorganisms [33]. The significant number of genes associated with signal transduction and membrane transport indicates that *B. amyloliquefaciens* LJ1 can sense its environment and adapt its metabolic pathways, enabling it to compete in changing environments [34]. The presence of genes implicated in protein biosynthesis and cellular structural components enables it to maintain healthy living state that contributes to its survival and biocontrol function [35]. Based on this study, the gene for secondary metabolite biosynthesis is significant because it may creates specific agents that can act as a natural antibiotic against several pathogen species in agriculture system. Therefore, the presence of a complex and interconnected set of genes gives it the power to act against other species in the community. Overall, the comprehensive functional annotation of *B. amyloliquefaciens* LJ1 provides a solid foundation for future studies aimed at elucidating the specific mechanisms underlying its beneficial effects and for exploiting its potential as a sustainable biocontrol agent.

### 3.4. Genomic Metabolic System Analysis

**Figure 4:**
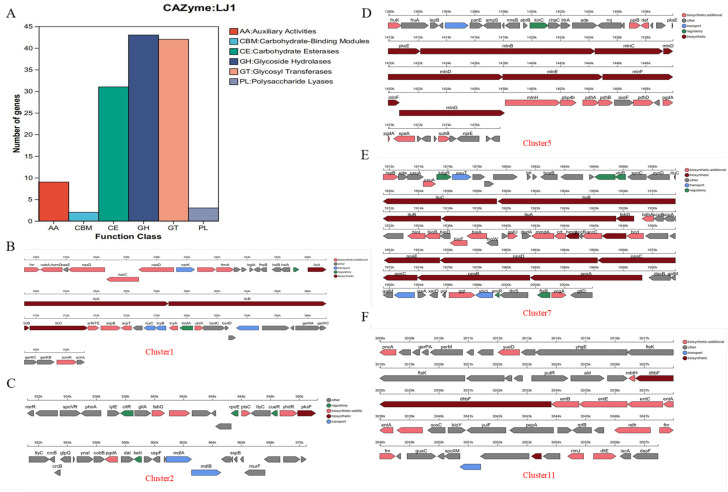
*B. amyloliquefaciens* LJ1 genomic metabolic system. (**A**) Functional classification diagram of carbohydrate active enzymes (CAZy); (**B**) Secondary metabolite synthesis gene cluster: Cluster1; (**C**) Secondary metabolite synthesis gene cluster: Cluster2; (**D**) Secondary metabolite synthesis gene cluster: Cluster5; (**E**) Secondary metabolite synthesis gene cluster: Cluster7; (**F**) Secondary metabolite synthesis gene cluster: Cluster11.

Analysis of the *B. amyloliquefaciens* LJ1 genome revealed a diverse array of CAZy and secondary metabolite biosynthetic gene clusters, providing insights into its metabolic capabilities and potential role in biocontrol. CAZyme analysis identified a total of 130 CAZyme-encoding genes, classified into six categories: GHs, GTs, PLs, CEs, CBMs, and AAs (Figure 4A). The most abundant categories were GHs (43 genes) and GTs (42 genes), followed by CEs (31 genes), indicating a versatile capacity for degrading and utilizing various carbohydrates in the environment [36]. The abundance of GHs suggests a strong ability to break down complex polysaccharides, such as cellulose, hemicellulose, and chitin, which are common constituents of plant cell walls and fungal mycelia [37]. This capability may contribute to the biocontrol activity of *B. amyloliquefaciens* LJ1, enabling it to degrade fungal cell walls and compete with plant pathogens for nutrients.

Secondary metabolite biosynthetic gene cluster analysis using antiSMASH identified 12 clusters located on the chromosome (Table 3 and Figure 4). These clusters encode various types of secondary metabolites, including non-ribosomal peptides (NRPs), polyketides (PKs), terpenes, and thiopeptides. Several clusters showed high similarity to known antimicrobial compounds, strongly suggesting that *B. amyloliquefaciens* LJ1 utilizes these compounds as a key mechanism for biocontrol. Among these, Cluster 1 showed 82% similarity to surfactin, Cluster 7 showed 100% similarity to fengycin, Cluster 11 showed 100% similarity to bacillibactin, and Cluster 12 showed 100% similarity to bacilysin. Surfactin and fengycin are well-known lipopeptides exhibiting strong antifungal activity by disrupting fungal cell membranes [38]. Bacillibactin is a siderophore that chelates iron, limiting its availability to plant pathogens and thereby inhibiting their growth [39]. Bacilysin is a dipeptide antibiotic that inhibits bacterial cell wall synthesis [40]. Other clusters, showing high similarity to macrolactin H (Cluster 5, 100% similarity) and difficidin (Cluster 10, 100% similarity), also exhibit potent antimicrobial activity [41]. The presence of terpene and thiopeptide clusters suggests the potential to produce additional novel compounds involved in biocontrol [42]. In summary, the genomic analysis of metabolic systems indicates that *B. amyloliquefaciens* LJ1 possesses a diverse enzymatic repertoire and an array of secondary metabolite biosynthetic gene clusters, contributing to its biocontrol potential. Future research should focus on the expression and activity of these genes and their metabolites under different environmental conditions and assess their role in controlling plant diseases. Further functional analysis must focus on identifying key genes.

### 3.5. Genomic Pathogenic System Analysis

**Figure 5:**
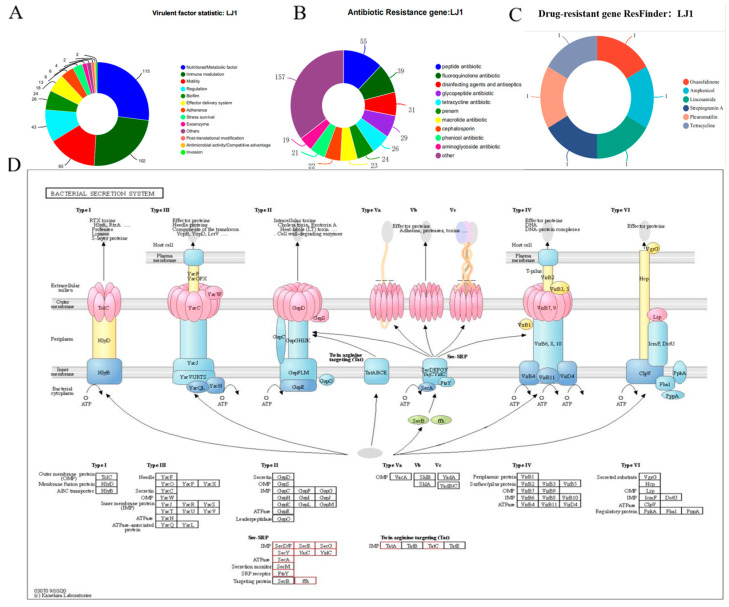
*B. amyloliquefaciens* LJ1 pathogenic system analysis. (**A**) Virulent factor statistic; (**B**) Antibiotic resistance gene statistic; (**C**) Prediction and classification statistics of drug resistance genes; (**D**) Secretory system pathway.

To assess the safety profile of *B. amyloliquefaciens* LJ1 for potential biocontrol applications, a comprehensive analysis was conducted to identify genes encoding virulence factors, antibiotic resistance determinants, and bacterial secretion systems. To assess the safety profile of *B. amyloliquefaciens* LJ1 for potential biocontrol applications, we performed a comprehensive genomic analysis, focusing on virulence factors, bacterial secretion systems, and antibiotic resistance genes. Analysis of the *B. amyloliquefaciens* LJ1 genome revealed a limited number of genes encoding putative virulence factors (Figure 5). The most abundant categories included Nutritional/Metabolic factors (115 genes), and Immune modulation (102 genes). These genes are useful for the competitive advantages of bacteria, but do not constitute virulence factors [11]. Further analysis identified genes encoding components of the Sec and Tat (twin-arginine translocation) secretion systems (Figure 5D). Notably, no genes encoding the type I, type II, type III, type IV, or type VI secretion systems were identified in the genome of *B. amyloliquefaciens* LJ1, which types of secretion are often related to virulence [43,44]. Moreover, the analysis of the *B. amyloliquefaciens* LJ1 genome identified a significant number of genes associated with antibiotic resistance (Figure 5C). The most abundant category was related to Glycopeptide antibiotic, followed by Peptide antibiotic. Further analysis using ResFinder (Figure 5A) revealed the presence of acquired resistance genes conferring resistance to Oxazolidinone, Amphenicol, Lincosamide, Streptogramin A, and Tetracycline, with each being detected only once in the genome.

The limited number of identified virulence factors and the absence of type III, IV, and VI secretion systems suggest that *B. amyloliquefaciens* LJ1 is unlikely to be a significant threat to plant or animal health. The presence of motility and biofilm-related genes allows it to compete in different settings [45]. However, as it relies on general secretion systems to move the protein, there is no transfer event to cause disease. The detection of antibiotic resistance genes raises concerns about the potential for horizontal gene transfer and the spread of antibiotic resistance in the environment. However, it is important to note that many of the identified antibiotic resistance genes may be intrinsic to *B. amyloliquefaciens* and may not necessarily confer resistance to clinically relevant antibiotics [46]. As the amount detected on the plasmid is low, *B. amyloliquefaciens* LJ1 is unlikely to transfer its resistance to other species. Overall, the genomic analysis provides a complex picture of the potential safety concerns associated with *B. amyloliquefaciens* LJ1.

### 3.6. Safety Evaluation

**Figure 6:**
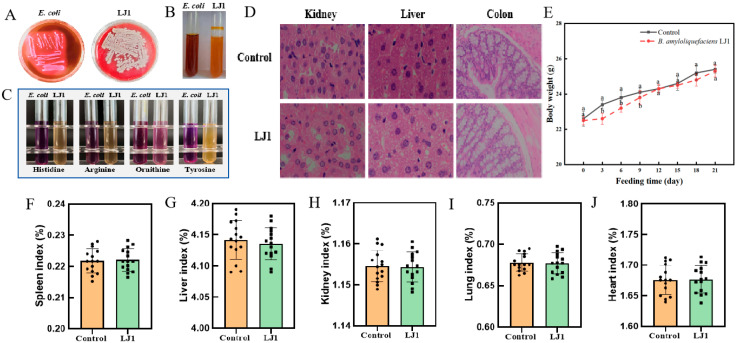
Safety evaluation and mice toxicology experiment of *B. amyloliquefaciens* LJ1. (**A**) Hemolysis experiment; (**B**) Indole test; (**C**) Amino acid decarboxylase experiment; The mice (**D**) organ slices, (**E**) body weight, and organ index analysis of (**F**) spleen, (**G**) liver, (**H**) kidney, (**I**) lung, (**J**) heart of feeding with *B. amyloliquefaciens* LJ1 for 21 day. Different lowercase letters indicate differ significantly at *p* < 0.05 by Duncan’s multiple range tests.

To comprehensively assess the safety profile of *B. amyloliquefaciens* LJ1, a series of in vitro and in vivo experiments were conducted to evaluate its metabolic characteristics, potential virulence factors, and effects on host physiology (Figure 6). In vitro safety assessments began with a hemolysis assay (Figure 6A). Hemolysins, pore-forming toxins or enzymes that lyse red blood cells, are often associated with bacterial virulence, potentially damaging host tissues and leading to anemia [47]. *B. amyloliquefaciens* LJ1 did not exhibit a zone of hemolysis on blood agar plates, indicating a lack of hemolytic activity. This suggests that *B. amyloliquefaciens* LJ1 does not produce such cytotoxic factors, contributing positively to its safety assessment. An indole test revealed that *B. amyloliquefaciens* LJ1 was indole-negative (Figure 6B). Indole production, typically resulting from tryptophan metabolism, is characteristic of some pathogenic bacteria and can enhance virulence by interfering with host cell function and exhibiting antimicrobial activity against beneficial bacteria [48]. The indole-negative phenotype further supports the benign nature of *B. amyloliquefaciens* LJ1. Amino acid decarboxylase assays demonstrated that *B. amyloliquefaciens* LJ1 was unable to decarboxylate histidine, arginine, or tyrosine (Figure 6C). The inability to decarboxylate these amino acids indicates that *B. amyloliquefaciens* LJ1 cannot produce corresponding biogenic amines via these pathways. The production of certain biogenic amines can be associated with food spoilage and potential health risks at elevated concentrations [49]. Therefore, the negative results of *B. amyloliquefaciens* LJ1 in these decarboxylase tests further strengthen its safety profile, suggesting that it does not produce these potentially harmful compounds.

The in vivo safety of *B. amyloliquefaciens* LJ1 was evaluated through a 21 day mice-feeding study. Body weight monitoring revealed no significant differences between the control group and the *B. amyloliquefaciens* LJ1-fed group (Figure 6E). Both groups of mice exhibited normal growth curves, suggesting that *B. amyloliquefaciens* LJ1 administration had no adverse effects on overall growth, feed intake, or metabolic health. Histopathological examination of liver, kidney and colon tissue sections revealed no observable abnormalities in the *B. amyloliquefaciens* LJ1-treated group compared to the control group (Figure 6D). The cellular architecture of hepatocytes, nephrocyte and the intestinal lining appeared normal, with no evidence of inflammation, necrosis, or structural damage. This is a key indicator that *B. amyloliquefaciens* LJ1 does not induce tissue-specific toxicity or inflammation. Furthermore, organ indices (spleen, liver, kidney, lung, and heart) for all assessed organs in the LJ1-fed group were comparable to those of the control group. This suggests that *B. amyloliquefaciens* LJ1 did not cause organ enlargement or reduction, which are common indicators of toxicity or immune response activation or organ damage [50].

Collectively, the in vitro and in vivo safety assessments provide robust evidence for the safety of *B. amyloliquefaciens* LJ1 as a potential biocontrol agent. The absence of hemolytic activity and indole production, along with the negative results for key amino acid decarboxylase activities, collectively indicate that *B. amyloliquefaciens* LJ1 lacks common bacterial virulence mechanisms and does not produce harmful biogenic amines through these specific pathways. Crucially, the comprehensive in vivo study demonstrated a lack of adverse effects on mice. The consistent growth curves and normal organ histology and indices strongly suggest that *B. amyloliquefaciens* LJ1 is well-tolerated, does not induce systemic toxicity, inflammation, or significant immunological responses that would alter organ development or function. These findings are critical for regulatory approval and consumer confidence in products containing *B. amyloliquefaciens* LJ1. The lack of detrimental impact on host health, coupled with its known beneficial properties, strengthens the argument for its suitability as a safe and effective biocontrol agent.

### 3.7. Biocontrol Activity of Bacillus amyloliquefaciens LJ1 Against Penicillium expansum

**Figure 7:**
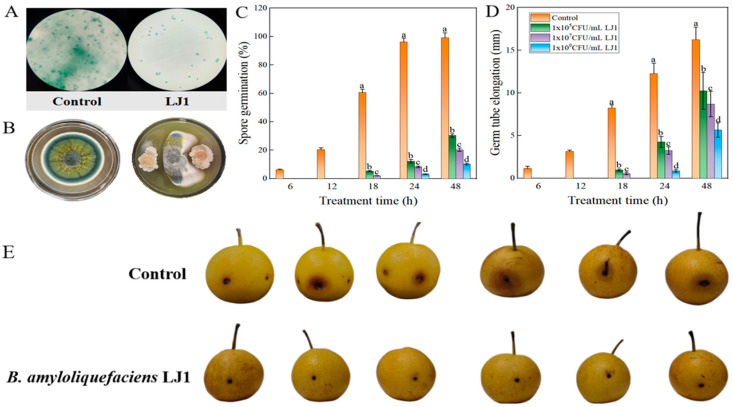
The inhibitory effect of *B. amyloliquefaciens* LJ1 on the growth of *P. expansum* in vitro. (**A**) Microscopic observation of spore germination of *P. expansum* for 24 h; (**B**) The 72 h inhibition zone size of *B. amyloliquefaciens* LJ1 against *P. expansum*; The Effect of *B. amyloliquefaciens* LJ1 on (**C**) spore germination and (**D**) germ-tube elongates of *P. expansum* at 25 °C. (**E**) Effect of *B. amyloliquefaciens* LJ1 pretreatment on the *P. expansum* disease incidence of Nanguo pear. Different lowercase letters indicate differ significantly at *p* < 0.05 by Duncan’s multiple range tests.

To assess the biocontrol potential of *B. amyloliquefaciens* LJ1 against fungal pathogens, its inhibitory effects on the growth of *P. expansum*, a significant post-harvest rot pathogen, were evaluated in vitro (Figure 7).

Microscopic observation after 24 h of incubation revealed a stark contrast between the control and *B. amyloliquefaciens* LJ1-treated samples (Figure 7A). In the control, *P. expansum* spores showed robust germination and extensive germ-tube elongation, forming a dense network. In contrast, samples treated with *B. amyloliquefaciens* LJ1 exhibited significantly reduced spore germination and severely inhibited germ-tube development, with many spores appearing dormant or having failed to grow. The assessment of inhibition zone size after 72 h of incubation (Figure 7B) provided further quantitative evidence of *B. amyloliquefaciens* LJ1’s antifungal activity. A clear zone of inhibition was observed around the colonies of *B. amyloliquefaciens* LJ1 when co-cultured with *P. expansum*, indicating the production of diffusible inhibitory substances or direct competition mechanisms.

Quantitative analysis of spore germination (Figure 7C) demonstrated that *B. amyloliquefaciens* LJ1 significantly inhibited *P. expansum* spore germination in a dose dependent manner. In the control group, spore germination reached nearly 100% by 18 h and remained high at 48 h. However, treatments with *B. amyloliquefaciens* LJ1 at concentrations of 1 × 10^5^, 1 × 10^7^, and 1 × 10^9^ CFU/mL resulted in drastically reduced germination rates, ranging from approximately 5–20% at 18 h, while higher concentrations showing more pronounced inhibition. Similarly, germ-tube elongation was severely suppressed by *B. amyloliquefaciens* LJ1 (Figure 7D). The control group showed substantial germ-tube growth, exceeding 15 mm by 48 h. In contrast, *B. amyloliquefaciens* LJ1 treatments at all tested concentrations significantly inhibited germ-tube elongation. At 24 h, *B. amyloliquefaciens* LJ1 treatments reduced elongation to below 5 mm. The higher the concentration of the *B. amyloliquefaciens* LJ1, the more significant the inhibitory effect.

The results strongly support the efficacy of *B. amyloliquefaciens* LJ1 as a biocontrol agent against the fungal phytopathogen *P. expansum*. The combined effects of inhibiting spore germination and suppressing germ-tube elongation are critical for preventing fungal infection [51]. *B. amyloliquefaciens* LJ1’s ability to drastically reduce germination from nearly 100% to less than 10% and inhibit germ-tube growth to minimal levels effectively halts the initial stages of fungal infection. The dose-dependent inhibition observed in both spore germination and germ-tube elongation suggests that *B. amyloliquefaciens* LJ1 produces potent antifungal compounds or employs effective competitive mechanisms that become more pronounced at higher cell densities. This is consistent with the genomic annotation identifying a significant number of genes involved in the biosynthesis of secondary metabolites, which are often responsible for the antagonistic activity of *Bacillus* species against phytopathogens [52]. These secondary metabolites could include lipopeptides, polyketides, or enzymes that directly disrupt fungal cell membranes, inhibit key metabolic pathways, or chelate essential nutrients [53]. The observed inhibition zone in Figure 7B further corroborates the production of diffusible antagonistic substances by *B. amyloliquefaciens* LJ1. This characteristic is highly desirable for a biocontrol agent, as it allows for effective action against the pathogen even at a distance. The combined evidence from microscopic observations, quantitative germination assays, germ-tube elongation measurements, and inhibition zone assays unequivocally demonstrates *B. amyloliquefaciens* LJ1’s significant potential to control *P. expansum* rot diseases.

## 4. Conclusions

This study successfully characterized the endophytic bacterium *B. amyloliquefaciens* LJ1 isolated from Nanguo pear fruit, providing a comprehensive understanding of its genomic features, safety profile, and biocontrol potential. The whole-genome sequencing revealed a genome rich in genes associated with carbohydrate metabolism and secondary metabolite biosynthesis, including clusters encoding well-known antifungal lipopeptides such as surfactin and fengycin, as well as siderophores like bacillibactin. Importantly, our multifaceted safety assessments, encompassing in vitro hemolysis, indole production, biogenic amine decarboxylation assays, and a 21 day mice-feeding trial, conclusively demonstrated that *B. amyloliquefaciens* LJ1 is non-pathogenic and well-tolerated, exhibiting no adverse effects on animal physiology or tissue integrity. Furthermore, in vitro experiments confirmed the potent antagonistic activity of *B. amyloliquefaciens* LJ1 against *P. expansum*, a critical post-harvest pathogen, by effectively inhibiting spore germination and germ-tube elongation. These findings collectively highlight *B. amyloliquefaciens* LJ1 as a robust and safe candidate for the development of biological control strategies against fungal diseases in Nanguo pear and potentially other agricultural crops. It is important to acknowledge that field or post-harvest application may impact non-target organisms and the native microbiome. Future studies should assess these potential ecological effects, as well as ensure compliance with relevant environmental regulations for biopesticides like those in the European Union.

## Figures and Tables

**Table 1:** Genome assembly results.

Parameter	Value
Genome Size (bp)	3,947,365
Chrom No.	1
Plas No.	0
GC Content (%)	46.48
CDS No.	3757
tRNA No.	95
rRNA No.	30

**Table 2:** Genome prediction results.

Type	Parameter	Value
Coded sequence	Gene No.	3757
	Gene Total Len (bp)	3,485,559
	Gene Average Len (bp)	927.75
	Gene Density (kb)	0.95
	GC Content in Gene Region (%)	47.32
	Gene/Genome (%)	88.30
	Intergenetic Region Len (bp)	461,806
	GC Content in Intergenetic Region (%)	40.17
tRNA	tRNAs No.	95
	Type of tRNAs No.	20
rRNA	rRNAs No.	30
	16S rRNA	10
	23S rRNA	10
	5S rRNA	10
House-keeping gene	House-keeping Gene No.	31
sRNA	sRNA No.	81
	In Genome (%)	0.2966
Tandem repeat	Repeat No.	70
	In Genome (%)	0.43
Interspersed repeat	SINE No.	16
	LINEs No.	25
	LTR No.	2
	DNA Transposon No.	7

**Table 3:** Secondary metabolite synthesis gene cluster results.

Location	Cluster ID	Type	MIBiG Accession	Similar Cluster	Similarity (%)	Gene No.
Chromosome	cluster1	NRPS	BGC0000433	surfactin	82	40
Chromosome	cluster2	PKS-like	BGC0000693	butirosin A/butirosin B	7	41
Chromosome	cluster3	terpene	-	-	-	23
Chromosome	cluster4	lanthipeptide-class-ii	-	-	-	30
Chromosome	cluster5	transAT-PKS	BGC0000181	macrolactin H	100	44
Chromosome	cluster6	transAT-PKS	BGC0001089	bacillaene	100	44
Chromosome	cluster7	NRPS	BGC0001095	fengycin	100	63
Chromosome	cluster8	terpene	-	-	-	22
Chromosome	cluster9	T3PKS	-	-	-	50
Chromosome	cluster10	transAT-PKS	BGC0000176	difficidin	100	40
Chromosome	cluster11	NRPS	BGC0000309	bacillibactin	100	45
Chromosome	cluster12	other	BGC0001184	bacilysin	100	42

## Data Availability

The original contributions presented in this study are included in the article. Further inquiries can be directed to the corresponding author.
